# Does Widespread Use of Hydroxychloroquine Reduce the Transmissibility of SARS-CoV-2 / COVID-19? An Ecological Correlational Study

**DOI:** 10.2174/1871526523666230522114836

**Published:** 2023-09-23

**Authors:** Fabricio Souza Neves

**Affiliations:** 1 Department of Internal Medicine, Health Sciences Center, Federal University of Santa Catarina (UFSC), Florianópolis, Brazil

**Keywords:** COVID-19, SARS-CoV-2, hydroxychloroquine, chloroquine, antiviral agents, basic reproduction number, epidemiology

## Abstract

**Background:**

At the beginning of the coronavirus disease (COVID-19) pandemic, hydroxychloroquine (HCQ) was widely used as a possible antiviral agent. Current knowledge indicates that HCQ has little or no effect on individual clinical outcomes of COVID-19, but populational effects on disease transmissibility are still unknown.

**Objective:**

This study investigates the hypothesis that massive HCQ consumption by a population may contribute to reducing the transmissibility of SARS-CoV-2 and COVID-19 spread by reducing the viral load of infected individuals.

**Methods:**

Public database of seven states from Brazil in 2020 were assessed, before the start of COVID-19 vaccination. The daily values of the COVID-19 effective reproduction number (Rt) were obtained. Associations between Rt values and the proposed predictor variables (prevalence of COVID-19 as a marker of collective immunity; social isolation indices; consumption of HCQ) were tested using multiple linear regression analysis.

**Results:**

In all seven states, consumption of HCQ was a significant negative predictor of Rt (β ranged from -0.295 to -0.502, *p* = 0.001). Furthermore, the mean derivative of Rt during the declining period of the COVID-19 incidence (the mean rate of variation) was also significantly negatively related to the mean HCQ consumption in that period (R^2^ = 0.895; β = -0.783; *p* = 0.011), meaning that the higher the HCQ consumption, the faster the decline of COVID-19 Rt. It suggests a dose-response phenomenon and a causal relationship in this association.

**Conclusion:**

The results of this study are compatible with the hypothesis that HCQ has small but significant *in vivo* antiviral effects that are able to reduce SARS-CoV-2 transmissibility at the populational level.

## 1 INTRODUCTION

Coronavirus disease (COVID-19), caused by severe acute respiratory syndrome coronavirus 2 (SARS-CoV-2), emerged in December 2019 in Wuhan, China [1], and the outbreak was declared a public health emergency of international concern by the World Health Organization on January 30, 2020. In response to the fast spread of this aerial-transmitted infectious disease without any known treatment, many countries imposed obligatory social distancing policies, including severe lockdowns, as an attempt to reduce the transmissibility rate of the disease (the effective reproduction number, Rt) to “flatten the curve” of cases and deaths, and to avoid the overwhelming of health care systems [2]. However, these policies also imply adverse effects on public health by leading to severe psychological, economic, and social impairments, so an urgent need for an effective treatment was set. Repurposing existing medicines was an initial strategy, and hydroxychloroquine or chloroquine (in this study, unless specified, these drugs will be referred to indistinctly as HCQ) were widely indicated as possible agents for treatment and prophylaxis against COVID-19, leading to an unprecedented worldwide medical controversy. Some claimed the rationale for HCQ use based on its antiviral and anti-inflammatory properties [3], and others were against it based on the low level of existing clinical evidence supporting its use. An emergency use authorization for HCQ against COVID-19 was issued by the Food and Drug Administration (FDA) in the U.S. in March 2020 and posteriorly revoked in June 2020 [4].

Current knowledge indicates that HCQ has little or no effect on the treatment of COVID-19, and it was recommended that ongoing trials about it should be stopped [5]. In fact, these poor results could even be predicted due to the pharmacokinetic properties of these drugs, which make them typical slow-acting agents. Antiviral effects of HCQ, if any, must be quite small in an acute illness and would be very difficult to detect clinically [6, 7]. Unfortunately, large and high-quality studies that could verify its effects on long-term prophylaxis or at the populational level were halted or became unfeasible [8].

A small antiviral effect of HCQ reducing viremia *in vivo* that may reduce the transmissibility of the disease (leading to a collective benefit, which can only be appreciated in population studies) would be compatible with the finding of reductions of the transmissibility of COVID-19 in susceptible populations. This reduction should be directly proportional to the consumption of HCQ in these populations.

Conditions that allow verifying this hypothesis occurred in Brazil during the first wave of the COVID-19 pandemic in 2020. In Brazil, the largest country in South America, the Federal Government declared a national public health emergency on February 3, 2020, due to the COVID-19 outbreak [9]. Brazil is a federative republic formed by different states. A central organization of the national public health system is called the “Sistema Único de Saúde” (Unified Health System - SUS). Still, the states have some autonomy, and for epidemiological purposes, each state is responsible for registering the cases of COVID-19 in its territory. Brazilian states have different population, geographic, economic, and social characteristics. They are grouped into five major regions by similarity: North (seven states), Central-West (three states and the Federal District), Northeast (nine states), Southeast (four states), and South (three states). All these states implemented social distancing laws early during the pandemic, in the second half of March 2020. Also, in the first wave of the COVID-19 pandemic in Brazil, early treatment protocols recommending the off-label use of HCQ as the main therapeutic agent were disseminated by private medicine groups and incorporated by the Brazilian Federal Ministry of Health [10]. However, most academic and public health authorities in Brazil recommended against using and distributing these medications through the public health system (SUS). This opposition was due to the lack of positive evidence in clinical trials regarding HCQ treatment of COVID-19 cases and concerns that focusing on treatment could result in inappropriate relaxation of social distancing measures. In Brazil, private health establishments can operate independently as complementary to the public health system, guaranteed by the Federal Constitution (Article 199), which also establishes the government's obligation to maintain the public health system [11]. Therefore, due to the opposite points of view (social isolation as a public health measure versus pharmacological outpatient treatment as a complementary option assumed by private practice), HCQ was widely prescribed by doctors acting as liberal professionals but was rarely prescribed by SUS physicians. Access to these medications occurred mainly through direct purchases in commercial pharmacies, not public distribution. Thus, in the Brazilian states where a larger part of the population can access the private practice of medicine and can acquire medicines in commercial pharmacies at their own expense, there was a variable large-scale use of HCQ during the first wave of the COVID-19 pandemic. In addition, during this period, all HCQ sales were required to be recorded in a publicly accessible national database. There was also a publicly available national database about social isolation based on mobile phone geolocation. It is worth mentioning that the Brazilian states in which the population has a greater purchasing power to acquire HCQ and mobile phones also have the best public systems for reporting and recording communicable diseases, so they are the states with the most reliable epidemiological data. Furthermore, in 2020, vaccination against COVID-19 was not yet available and could not confound the results of disease transmissibility. Thus, all data necessary to test the hypothesis of this study was publicly available in Brazil.

The aim of this study was to verify if a correlation is found between the consumption of HCQ and the reduction of the transmissibility of COVID-19, measured by the effective reproductive number Rt (the number of people infected from a single case of COVID-19), controlling for the prevalence of COVID-19 (as a marker of collective immunity), and for the social isolation practiced by the population of the seven Brazilian states that belong to the South and Southeast regions (Espírito Santo, Minas Gerais, Rio de Janeiro, and São Paulo from the Southeast region plus Paraná, Rio Grande do Sul and Santa Catarina from the South region), during the first wave of COVID-19 pandemic, in 2020.

## 2 MATERIALS AND METHODS

An observational, retrospective, analytical, ecological, and correlational, study was performed based on secondary data obtained from public databases of the seven Brazilian states that form the South and Southeast Brazilian regions, covering the period from March 1, 2020, to October 13, 2020. These states were grouped because they share similar geographical, demographic, and economic characteristics. They comprise a population of nearly 120 million inhabitants [12] and their average household per capita income is 50% higher than the average of all other Brazilian states [13].

In Brazil, COVID-19-related severe acute respiratory syndrome cases and deaths are reported to the national electronic reporting system. Data on all suspected and confirmed COVID-19 cases are publicly available on the Brazilian COVID-19 portal [14]. In this study, data on severe acute respiratory syndrome cases due to confirmed COVID-19 were obtained from the analysis of the complete spreadsheet (CSV file) of the National Severe Acute Respiratory Syndrome Database, which was accessed on November 16, 2020 [15]. The cases retrieved from this file were included only if the variable ‘final case classification’ (CLASSI_FIN) was defined as ‘due to COVID-19’ (value ‘5’). Based on the variable ‘Acronym of the Federation Unit’ (SG_UF_NOT), the cases in each state were organized in chronological order, according to the variable ‘Date of the First Symptoms’ (DT_SIN_PRI) [16].

To allow accurate comparisons between each state’s results, only data on severe acute respiratory syndrome cases resulting from confirmed SARS-CoV-2 were considered as the marker of the incidence of COVID-19 cases, and not the total number of outpatient COVID-19 plus hospitalized COVID-19 cases. It allows for avoiding the bias induced by the different underreporting rates of outpatient COVID-19 cases in different Brazilian states [17]. Severe acute respiratory syndrome is treated and notified in hospitals, and in the period of this study, the analyzed Brazilian states did not have their health systems overwhelmed by the pandemic; thus, the registry of the number of severe acute respiratory syndrome due to COVID-19 is the most reliable marker of the incidence and prevalence of the disease for comparison between different Brazilian states.

Based on the daily number of COVID-19 new severe acute respiratory syndrome as defined, the daily values of the effective reproduction number (Rt) of COVID-19 were obtained by the online publicly available Epidemiologic Calculator developed by the University of Brasília [18]. To correct the effect of variations in notifications on weekend days, in this study, the values of the 7-day moving average were considered as the results of daily COVID-19 incidence and daily Rt.

The prevalence of COVID-19 adopted as a marker of collective immunity against the disease, was also calculated from the data on severe acute respiratory syndrome cases due to COVID-19 [15]. The sum of all cases of severe acute respiratory syndrome cases due to COVID-19 from the beginning of the pandemic to a given date, normalized according to the population of the state [12], was considered as the prevalence of the disease in the state on that date.

The social isolation indices in the Brazilian states were obtained from the ‘Brazilian COVID-19 Map’ (2020 Inloco©, Recife, Brazil) [19]. According to the company, data on individuals’ locational behaviours were obtained through a network of mobile apps; after user consent, the user’s location information was made available to the company anonymously and aggregated. The company data bank at that time comprised data on approximately 60 million Brazilian cell phone users. The social isolation index was calculated as the number of users in a region that did not leave their place of residence on a given day in relation to the total number of users in the same region. Also for this variable, the values of the 7-day moving average were considered as the results of the daily social isolation index.

The number of units (pills) of HCQ sold for outpatient use in the Brazilian states was obtained by consulting the National System for the Management of Controlled Products of the National Health Surveillance Agency [20]. The system provides data about the number of units of a particular drug sold per month for each Brazilian state. For each Brazilian state, the total number of units sold (the sum of pills) of all hydroxychloroquine and chloroquine formulations (in this study, we used the acronym HCQ interchangeably to name this set of medications) was recorded for each month. The daily number of units sold was estimated by dividing the number of units sold in the month by the number of days. Data on daily HCQ sales were normalized according to the state population estimated in July 2020 [12]. For this variable, the values of the 30-day moving average were considered as the results of daily HCQ sales.

The first analysis to test this study’s hypothesis was made by multiple linear regressions, in each Brazilian state, between COVID-19 Rt as the outcome and daily social isolation index, HCQ consumption, and COVID-19 prevalence as the predictors, in simultaneous entry. This first analysis may reveal a correlation between outcome and predictors, but being an observational study, it could not define the cause-effect relationship. A significant relationship could be explained in both ways, as higher HCQ consumption being stimulated by the growing number of COVID cases without influencing disease transmissibility (HCQ consumption being a mere consequence of the natural increase and decrease of COVID-19 transmissibility and incidence) or the opposite (increased HCQ consumption leads, after a certain point, to the reduction in the incidence of COVID-19 by reducing the transmissibility of the disease).

To overcome this limitation, the second analysis in the present study was based on the mean derivative of the Rt curve in the period of reduction of Rt (the “declining period” of the pandemic) around the inflection point of the incidence of COVID-19. The declining period of the pandemic was defined in each Brazilian state as the period starting in the day with the maximum Rt value followed by a continuous decline in Rt until the day with the minimum Rt value followed by a new continuous rising in Rt value, both occurring at least one month after the start of social isolation measures imposed in all states at the end of March.

The mean derivative of Rt (the mean rate of variation) is a marker of the “velocity” of the decrease of the wave of the pandemic and can be determined as (being Dt = one day):

mean dRt = [(ΔRt1/Δt1) + (ΔRt2/Δt2) … + (ΔRtn/Δtn)]/n

Mean dRt is related to the linear slope of the curve of Rt in the defined period of analysis. More negative values of dRt mean a faster decline in the pandemic. Thus, if mean dRt is inversely related to mean HCQ consumption in the declining period (the higher the HCQ consumption, the faster the decline of Rt with more negative dRt), it is a piece of evidence in favour of the hypothesis of an antiviral effect of HCQ reducing COVID-19 transmissibility. Oppositely, if mean dRt is positively related to HCQ consumption (the slower the decline of Rt with less negative dRt, the higher the HCQ consumption) is evidence of HCQ consumption merely as a consequence of COVID-19 cases (more COVID-19 cases stimulating the use of HCQ without HCQ effect on COVID-19 transmissibility).

The data were compiled in Excel 365 spreadsheets (Microsoft©, Redmond, USA). Statistical analyses were performed using IBM SPSS Statistics for Windows (version 27.0; IBM Corp., Armonk, USA). This study used only anonymized secondary data from publicly available databases, according to Federal Law 12,527 (November 18, 2011) being therefore free from approval by the Ethics Committee, as established by Resolution 510 (April 7, 2016) of the National Health Council of Brazil. There was not any financial support for this study.

## 3 RESULTS

Descriptive statistics are depicted in Fig. (**1a**-**g**). For each Brazilian state, two charts are shown: one with the outcome (daily Rt; daily COVID-19 incidence is also shown) and another with the proposed predictors (daily social isolation index, HCQ consumption, and COVID-19 prevalence). Two major decreases in Rt values can be observed in all Brazilian states. The first (grey arrows) is related to the increase in social isolation indices by the end of March. The second one occurs late in the graph and can be related to the increase in HCQ consumption (black arrows).

The first analysis to test this study’s hypothesis was made by multiple linear regressions, in each Brazilian state, between Rt as the outcome and daily social isolation index, HCQ consumption, and COVID-19 prevalence as the predictors with simultaneous entry. The results are shown in Table **1**. In all Brazilian states, the models with the three predictors were statistically significant, with R^2^ ranging from 0.589 (RS) to 0.955 (RJ). HCQ consumption is a significant predictor of Rt variability in all analyzed Brazilian states (p<0.001).

The second analysis performed to test the hypothesis of this study was a multiple linear regression between the mean dRt of the declining period of the pandemic of the seven analyzed Brazilian states as the outcome, and the mean HCQ consumption and the mean social isolation index in the same period as predictors. The declining periods of each state are shown in Fig. (**1**) (the beginning is marked with a black arrowhead, and the end is marked with a black arrow). In all Brazilian states, the declining period is temporally associated with increased HCQ consumption. It can be visually observed that the decline of Rt is faster (the curve of Rt is more negatively inclined) as higher HCQ consumption is during the declining period. Results of the analysis are shown in Table **2** and Fig. (**2**): the mean derivative of Rt during the declining period of the COVID-19 incidence (the mean rate of variation) is significantly related negatively to the mean HCQ consumption in that period (R^2^ = 0.895; β = -0.783; p = 0.011), meaning that the higher the HCQ consumption, the faster the decline of COVID-19 Rt.

## 4 DISCUSSION

In a randomised, double-blind, placebo-controlled clinical trial [21], open trials [22-24] and observational studies [25], the use of HCQ in the early treatment of COVID-19 reduced the viral load of SARS-CoV-2. However, in other open trials [26-28] and observational studies [29] there were no differences in nasopharyngeal viral clearance in groups treated with HCQ compared to controls. This subject remains controversial, and systematic reviews with meta-analysis have reported conflicting results, depending on the studies considered, with significant heterogeneity between them [30-32].

Theoretically, reducing SARS-CoV-2 viral load by HCQ treatment could improve the control of the pandemic in a population because the transmissibility of COVID-19 depends on the viral load around the onset of the disease [33]. Disease transmissibility in a population is an outcome that cannot be adequately evaluated in clinical trials. Epidemiological studies with data from health surveillance, observational, or interventional population-based studies are warranted to assess this outcome [34].

This is the first study that sought to correlate the rates of HCQ consumption with the transmissibility of COVID-19 comparing several different large populations. The findings in Table **1** strongly confirm the relationship between HCQ consumption and COVID-19 incidence previously observed in a smaller study based in one Brazilian state [35] and suggest that this relation is not casual because now it was significantly found in all seven states that were analysed. The findings in Table **2**, revealing a negative correlation between HCQ consumption and dRt (the higher the HCQ consumption, the faster the decline of the pandemic with more negative dRt), and in Fig. (**2**), depicting a “dose-response” phenomenon, suggests the causal relationship between these two variables being high HCQ consumption the predictor of faster declining in COVID-19 transmissibility. The dose-response effect, one of Hill´s criteria for causation [36], was also previously glimpsed in a clinical trial about HCQ on COVID-19 prophylaxis, but the study was not designed to observe it [37].

The main limitation of this study is the possibility of the occurrence of an “ecological fallacy”: it cannot be assured that the individuals infected with SARS-CoV-2 are in fact the users of the HCQ sold. However, this possibility could lead, in this type of study, to the non-detection of an existing correlation (type II error), in the case if HCQ had been used indiscriminately and on a large scale by people not infected with SARS-CoV-2. To spuriously induce the correlation found in this study, it would be necessary for some other factor capable of reducing the transmissibility of SARS-CoV-2 to be present in the scenario and also to be proportional to the consumption of HDQ. Vaccination against COVID-19, as already mentioned, was not available at the time of this study and could not have influenced the results. The use of masks could be a cause of confusion, but it can be observed that in four of the states analyzed, the mandatory use of masks began many months before the period of decline of the pandemic (Rio Grande do Sul, on May 10; [38] Santa Catarina, on April 17; [39] Paraná, on April 28; [40] and Minas Gerais, on April 17); [41] in one state, the mandatory use of masks began after the declining period (Rio de Janeiro, on June 3); [42] and in just two states (São Paulo, on May 7 [43] and Espírito Santo, on May 8) [44] the mandatory use of masks coincided with the period considered to be the decline of the pandemic. Also, there was also no reinforcement in non-pharmacological measures against transmission of COVID-19 in the declining periods of the pandemic analysed in this study. This is evident when observing the graphs of social isolation indices in Fig. (**1**). In all states, there is a significant increase in social isolation only at the time of the initial decrees on the subject (March 2020). What happened in Brazil was a slow relaxation and decline of all non-pharmacological measures to contain the pandemic, after their implementation, during the year 2020. Thus, it is very unlikely that the use of masks had been associated with the declining periods of the pandemic in the way found in this study.

The positive results of the present study are not contradictory to the negative results of HCQ in clinical trials on treatment and prophylaxis against COVID-19. The results of all studies are compatible with the pharmacologic characteristics of HCQ. HCQ may reduce the viral entry process of SARS-CoV-2 in human cells by alkalinization of endolysosomes [45]. However, due to its large volume distribution, reaching effective HCQ tissue concentrations is challenging within a few days of use [46]. Also, HCQ acts against one of the entry pathways of SARS-CoV-2 (the endosomal pathway) but not against the other (the cell surface pathway) [47]. Thus, the antiviral action of HCQ against SARS-CoV-2 is expected to be of small effect size and could only be appreciated by an outcome that is sensitive enough to reveal small variations proportionally to SARS-CoV viral load in infected individuals, as occurred in this study that used COVID-19 transmissibility as the main outcome. The antiviral effect size of HCQ as a single therapeutic agent is probably lower than the necessary cutoff to improve huge outcomes, such as mortality or hospitalizations.

The main consequence of admitting this study’s hypothesis would be recognizing HCQ in its real possibilities as an antiviral agent. As a single agent, HCQ is not an effective treatment or prevention for the disease at the individual level. Nevertheless, it could contribute to the control of the pandemic at the population level by reducing disease transmissibility; it could be used as a less expensive component of a multi-drug therapeutic antiviral scheme based on different mechanisms of action; and it can also serve as a model for the development of new antiviral drugs, with more favourable pharmacokinetic characteristics.

## CONCLUSION

The results of this study reinforce the hypothesis that HCQ consumption in large scale by a population reduces the transmissibility of COVID-19. It suggests that HCQ may have an antiviral effect against SARS-CoV-2 *in vivo* and could contribute as an auxiliary measure to control the COVID-19 pandemic.

## Figures and Tables

**Fig. (1):**
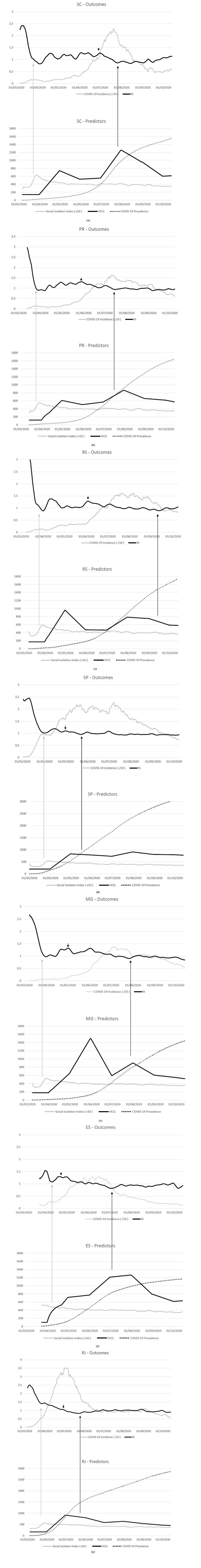
**a**) Santa Catarina (SC); **b**) Paraná (PR); **c**) Rio Grande do Sul (RS); **d**) São Paulo (SP); **e**) Minas Gerais (MG); **f**) Espírito Santo (ES); **g**) Rio de Janeiro (RJ). For each analysed Brazilian state, two charts are shown. The upper chart shows the outcome daily effective reproduction number (Rt). Daily COVID-19 incidence is also shown. The bottom chart presents the proposed predictors daily social isolation index (%), daily hydroxychloroquine consumption (pills per million inhabitants), and daily COVID-19 prevalence (cases per million inhabitants). Two major decreases in Rt values (when Rt became less than 1) can be observed in all Brazilian states. The first (grey arrows) is related to the increase in social isolation indices by the end of March. The second one occurs late in the graph during the declining period of the first wave of the pandemic. The beginning and the end of the analysed declining period is marked by black arrows.

**Fig. (2):**
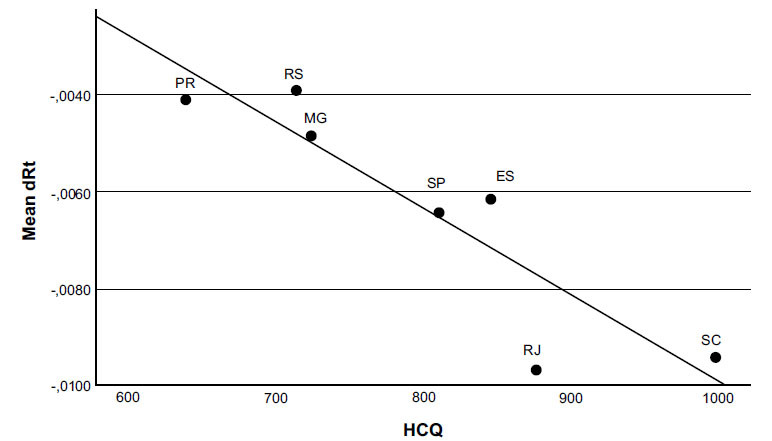
Chart of the mean derivative (mean rate of variation) of effective reproduction number of COVID-19 (mean dRt) as a function of the mean daily consumption of hydroxycloroquine (HCQ), expressed in pills per million inhabitants, during the declining period of the first wave of COVID-19 pandemic in the seven analysed Brazilian states: Santa Catarina (SC), Rio de Janeiro (RJ), Espírito Santo (ES), São Paulo (SP), Minas Gerais (MG), Rio Grande do Sul (RS), Paraná (PR).

**Table 1:** Multiple linear regressions with simultaneous entry of predictor variables (prevalence of COVID-19; social isolation index; consumption of hydroxychloroquine) for the outcome effective reproduction number of COVID-19 (Rt). South and Southeast Brazilian states, 2020.

**Outcome**	**States**	**Model Characteristics**			**Prevalence of COVID-19**		
		**F change**	**R^2^**	***p***	**β**	**t**	***p***
Rt	ES	262.2	0.796	<0.001	-0.407	-5.192	<0.001
	MG	300.0	0.806	<0.001	-1.016	-27.664	<0.001
	PR	220.9	0.758	<0.001	-0.716	-14.727	<0.001
	RJ	1540.5	0.955	<0.001	-0.903	-44.758	<0.001
	RS	103.3	0.589	<0.001	-0.523	-9.405	<0.001
	SC	218.8	0.751	<0.001	-0.488	-10.174	<0.001
	SP	603.4	0.890	<0.001	-0.683	-17.247	<0.001
**-**	**-**	**Social Isolation Index**			**Consumption of Hydroxychloroquine**		
**-**	**-**	**β**	**t**	***p***	**β**	**t**	***p***
-	ES	0.236	3.094	0.002	-0.375	-9.822	<0.001
-	MG	-0.852	-22.974	<0.001	-0.404	-13.200	<0.001
-	PR	-0.914	-20.640	<0.001	-0.502	-11.467	<0.001
-	RJ	-0.599	-30.358	0.062	-0.295	-16.595	<0.001
-	RS	-0.616	-12.027	<0.001	-0.417	-8.666	<0.001
-	SC	-0.739	-19.101	<0.001	-0.492	-11.070	<0.001
-	SP	-0.734	-26.902	<0.001	-0.326	-9,321	<0.001

**Table 2:** Multiple linear regressions with simultaneous entry of predictor variables (mean social isolation index; mean consumption of hydroxychloroquine) for the outcome mean derivative of the effective reproduction number of COVID-19 (Rt). South and Southeast Brazilian states, 2020.

**Outcome**	**Model Characteristics**		
**F Change**	**R^2^**	***p***
Mean derivative of Rt	17.097	0.895	0.011
**Predictors**			
-	**β**	**t**	***p***
Social isolation index	-0.311	-1.780	0.150
Consumption of hydroxychloroquine	-0.783	-4.477	0.011

## Data Availability

All data used in this study are publicly available in the sources quoted in the References list. To facilitate data checking, the file with the database of cases of acute respiratory syndrome in Brazil in 2020 and the work file for generating the descriptive results of this study are presented as supplementary files.

## LIST OF ABBREVIATIONS

COVID-19: Coronavirus Disease 19
FDA: Food and Drug Administration
HCQ: Hydroxychloroquine
SARS-CoV-2: Severe Acute Respiratory Syndrome Coronavirus 2
